# GSEA model outcomes in pharmaceutical workforce development: a retrospective pilot study (2023–2025)

**DOI:** 10.3389/fpubh.2026.1812820

**Published:** 2026-05-26

**Authors:** Shibao LI, Chenyang Ma

**Affiliations:** 1 School of Ecology and Environment, Tibet University, Lhasa, China; 2 Dalian Municipal Central Hospital, Dalian, China

**Keywords:** health human resources, multi-stakeholder governance, pharmaceutical industry, retrospective evaluation, workforce development

## Abstract

**Background:**

The pharmaceutical industry faces structural challenges, such as high compliance costs and rapid technological change, which hinder sustainable workforce development in small- and medium-sized cities. Traditional school–enterprise models often fail to address these constraints, leading to persistent misalignment between educational provision and industry needs. This study addresses both practical and theoretical gaps in workforce development for a highly regulated sector that is also integral to the public health infrastructure in resource-constrained settings.

**Objective:**

This retrospective evaluation examines a pilot government–school–enterprise–association (GSEA) intervention implemented from 2023 to 2025 and assesses its observed associations with pharmaceutical workforce development and industry–education integration. It also clarifies the model’s intended theoretical contribution, namely as a governance arrangement for translating public policy into professional standards and curriculum–practice alignment in a highly regulated industry.

**Methods:**

A retrospective evaluation of a pilot intervention was conducted in a prefecture-level city in eastern China. The GSEA model was implemented as a pilot program from January 2023 to December 2025, with formal ethical approval and a comprehensive assessment conducted in 2025. Data from routine program monitoring—including stakeholder surveys (*n* = 326), interviews (*n* = 28), and administrative panel data (*n* = 1,200 students)—were analyzed retrospectively using structural equation modeling (SEM) to examine governance mechanisms and their associations with talent development, school–enterprise cooperation, and industry service capacity.

**Results:**

The implementation of the GSEA model was associated with significant improvements in workforce alignment: the professional employment rate increased from 68.3 to 89.5%, and enterprise satisfaction with graduates rose by 23.6%. The observed improvements were also accompanied by model-consistent patterns, in which policy support was related to association-led standard translation, curriculum–practice alignment, and enterprise participation. Given the retrospective observational design, these findings are interpreted as associative rather than causal.

**Conclusion:**

The GSEA model offers a context-sensitive governance framework for workforce development in highly regulated industries. This model is particularly relevant in settings with sufficient policy coordination, intermediary capacity, industry participation, and minimum fiscal support. Future research should test the model using multi-site quasi-experimental and longitudinal designs.

## Introduction

The pharmaceutical industry, as a “livelihood-critical” and “technology-intensive” sector, is a strategic pillar for safeguarding national health and driving innovation-led development. It also serves as a core field for advancing the United Nations Sustainable Development Goal 3 (“Good Health and Well-being”) (1). The development of a skilled pharmaceutical workforce is essential for public health infrastructure, particularly in the Global South, where workforce shortages and skill mismatches constrain access to essential medicines and healthcare services. According to the 2024 Report on the Economic Operation of China’s Pharmaceutical Industry, the country’s pharmaceutical industry achieved a total output value of 4.8 trillion yuan in 2023, accounting for 4.1% of the GDP, with a compound annual growth rate (CAGR) of 5.4%—significantly outpacing the 2.8% average growth rate of traditional manufacturing (2). Among the different sub-sectors, chemical pharmaceuticals grew by 6.2%, biopharmaceuticals by 8.5%, and medical devices, by 7.3%, reflecting robust resilience in development (3).

The 14th Five-Year Plan for the Development of the Pharmaceutical Industry further mandates that by 2025, China’s pharmaceutical industry must achieve an internationally advanced level in innovation, quality, and green development, creating a demand for over 2 million new high-quality technical talents (4). As the primary source of skilled talent, pharmaceutical vocational education plays a pivotal supporting role. However, a structural “three highs and three lows” mismatch persists between the talent supplied by vocational education and the needs arising from industrial upgrading. On the one hand, industry demand for highly skilled, compliance-proficient, and interdisciplinary talents—particularly those with expertise in intelligent pharmaceutical equipment operation, drug compliance management, and pharmaceutical e-commerce—continues to surge (5). Data from a leading recruitment platform show that graduates with dual qualifications in “GMP certification + intelligent equipment operation” command a 35% salary premium. On the other hand, vocational college graduates show low professional employment rates (64.8%), low enterprise satisfaction (58.7%), and low industry skill certification pass rates (47.3)—all of which fall below industry expectations (6).

This mismatch stems from the traditional “school–enterprise dual” model’s failure to overcome the unique constraints of the pharmaceutical industry, which can be summarized in three key limitations: Low enterprise participation, fragmented cooperation, and Strict Good Manufacturing Practice (GMP) and Good Supply Practice (GSP) requirements, which impose additional “compliance costs” on enterprises when hosting student internships, including cleanroom renovations (exceeding 500,000 yuan per workshop), specialized safety training (2,000 yuan per person), and compliance risk mitigation (e.g., rework losses from drug contamination) (7). A 2023 survey by the National Medical Products Administration found that 72% of small- and medium-sized pharmaceutical enterprises refused school–enterprise cooperation due to “excessive compliance costs and no direct returns.” Consequently, collaboration remains superficial, often limited to “visit-based internships” or “short-term joint laboratory construction,” rather than establishing long-term, stable mechanisms (8). Intellectual property (IP) protection barriers and hindered technological cooperation. Pharmaceutical enterprises’ core competitiveness relies on IP, such as drug formulas and production processes. However, technological R&D and process optimization in school–enterprise cooperation pose significant risks of commercial secret leakage. A 2024 report by a provincial pharmaceutical industry association revealed that 68% of enterprises refused to share core technical data with colleges due to “fear of IP infringement.” Only 32% of cooperation projects involved substantive R&D efforts; the remaining projects were limited to “low-tech practical training.” As a result, colleges were unable to access cutting-edge industrial technologies, leading to curricula that are outdated compared to industry practices (9). Weak implementation of cooperation agreements and vague responsibility definitions. Traditional school–enterprise cooperation relies heavily on “verbal agreements” or “framework contracts,” with ambiguous clauses regarding internship assessment standards, enterprise instructors’ teaching responsibilities, and practical training equipment maintenance (10). A 2023 survey by the China Vocational and Technical Education Association found that 45% of projects were terminated early due to “enterprises failing to provide agreed internship positions” or “colleges missing skill training targets.” Without clear dispute resolution mechanisms, only 12% of conflicts were resolved through negotiation, eroding mutual trust (11).

To address these bottlenecks, scholars worldwide have explored multi-stakeholder involvement in industry–education integration. Internationally, Germany’s “dual system” leverages guilds to set vocational standards, with enterprises providing 70% of practical training. However, this model depends on high social welfare and large enterprise support, making it incompatible with China’s industrial structures in small- and medium-sized cities (dominated by small- and medium-sized pharmaceutical enterprises) (12, 13). The UK’s “apprenticeship system” uses government subsidies to incentivize enterprise participation but primarily focuses on general skills, lacking tailored mechanisms for the pharmaceutical industry’s compliance demands (14, 15). Domestically, studies have proposed a “government–school–enterprise–association” quadripartite collaboration model, but most of these proposals remain limited to qualitative role descriptions, lacking empirical testing of causal mechanisms (e.g., “how policies translate into standards” or “how standards guide teaching”). In addition, quantitative research in the pharmaceutical sector accounts for less than 5% of related studies, neglecting the impact of compliance constraints on cooperation (16, 17).

From a public policy perspective, workforce development in highly regulated sectors is not merely an educational design issue but also a governance problem involving institutional coordination, incentive alignment, and the translation of public rules into operational training standards. In this context, the government–school–enterprise–association (GSEA) model is understood here not simply as an expanded partnership structure but as a coordinated governance arrangement. In this model, the government provides policy incentives and convening authority, associations translate regulatory and occupational requirements into standards, colleges incorporate these standards into curricula, and enterprises provide practice settings and feedback loops (18–21).

Against this backdrop, this study implements a GSEA quadripartite collaborative framework in a prefecture-level city in eastern China with a developed pharmaceutical industry. Using mixed methods, this study addresses three questions: (1) Was the GSEA pilot associated with improvements in talent cultivation quality, school–enterprise cooperation efficiency, and industry service capacity? (2) Through what institutional mechanisms did the government, schools, enterprises, and associations interact within the model? (3) Under what enabling conditions might this governance arrangement be transferable to other highly regulated industrial contexts? Scientifically, the study clarifies the theoretical contribution of the GSEA model as a policy-to-standards-to-curriculum coordination framework for skills systems in regulated industries, rather than merely a broader variant of the traditional school–enterprise model. Practically, this study evaluates whether this arrangement can help align vocational training with local pharmaceutical workforce needs (22–24).

## Materials and methods

### Study design and setting

A retrospective evaluation of a pilot intervention was conducted in a prefecture-level city in eastern China that has a well-established pharmaceutical industry. The GSEA model was implemented as a pilot program from January 2023 to December 2025 under the oversight of the Municipal Bureau of Education and the Municipal Bureau of Industry and Information Technology. Routine administrative indicators were generated during pilot implementation, whereas the integrated research evaluation, including surveys, interviews, and SEM analyses, was completed in 2025.

The pilot program involved 2 municipal vocational colleges (offering 6 pharmaceutical-related majors, with 1,200 enrolled students), 12 local pharmaceutical enterprises (4 in production, 5 in distribution, and 3 in retail), the Municipal Bureau of Education, the Municipal Bureau of Industry and Information Technology, and the Municipal Pharmaceutical Industry Association. Data collection during the pilot phase (2023–2025) was conducted as part of routine program monitoring and administrative record-keeping, with formal research evaluation and ethical approval completed in 2025. (Table 1).

**Table 1:** Implementation timeline and core components of the GSEA pilot (2023–2025).

Phase/year	Lead actors	Core implementation focus	Nature of component
2023 (Pilot launch)	Government, colleges, and enterprises	Policy coordination, internship expansion, baseline curriculum revision, and establishment of joint meetings	Core pilot arrangement
2024 (Mechanism consolidation)	Association, colleges, and enterprises	Regional competency standards, teacher enterprise training, and broader project-based cooperation	Core pilot arrangement
2025 (Evaluation and optimization)	All four actors	Routine monitoring consolidation, mixed methods evaluation, and refinement of curriculum–practice alignment	Evaluation and optimization stage

### GSEA collaborative framework design

A localized GSEA framework was developed to clarify the core responsibilities of each stakeholder:

Government: Provided policy support (1,000 yuan/student/year subsidy for enterprises hosting interns, 15% tax credit for collaborative R&D projects), established an interdepartmental coordination team (monthly joint meetings of the Education Bureau, Industry and Information Technology Bureau, and Market Supervision Bureau), and monitored pilot progress (quarterly performance bulletins) (25).

Vocational colleges: Revised curricula based on industry feedback (adding courses such as Intelligent Pharmaceutical Equipment Operation, Drug Compliance Management, and Pharmaceutical E-commerce Operations; updating syllabi for three core courses), mandated enterprise-based practical training for teachers (2 months/year, with qualification tied to promotion), and undertook enterprise-commissioned R&D projects (≥5 projects/year) (26).

Enterprises: Provided ≥300 internships/year (covering production, quality control, and sales), seconded 15 + technical experts for part-time teaching (≥120 teaching hours/year), and donated practical training equipment (valued at ~1 million yuan, including intelligent decocting machines and drug traceability systems) (27).

**Figure 1:**
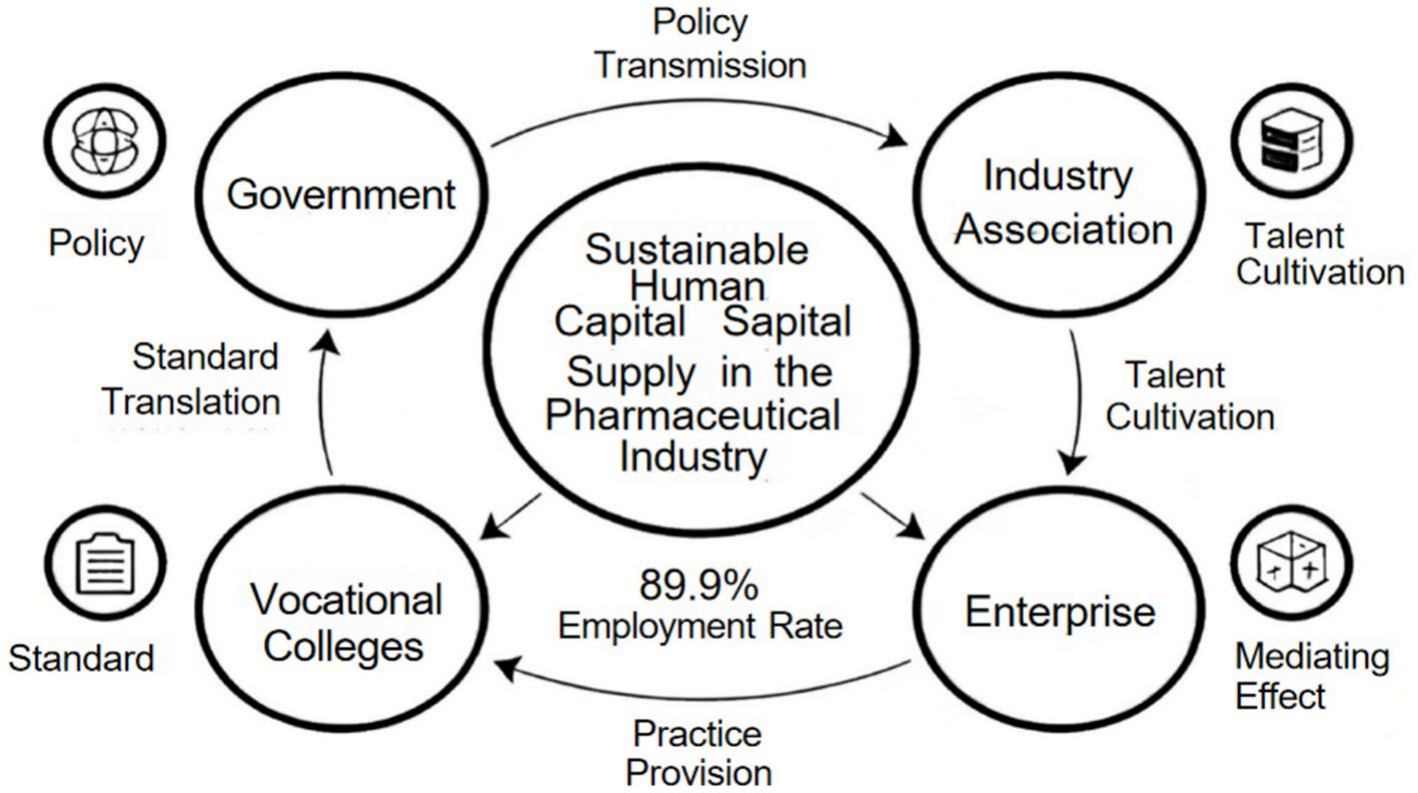
Sustainable human capital supply in the pharmaceutical industry. A matrix illustrating cross-stakeholder responsibilities. The government leads policy support (subsidies and tax credits), interdepartmental coordination, and pilot supervision. Vocational colleges lead curriculum revision, teaching, and teacher enterprise training.

Industry association: Developed three regional vocational competency standards (for pharmaceutical production technicians, pharmaceutical e-commerce operators, etc.), organized biannual skill competitions (aligned with job competency standards), and published annual Pharmaceutical Industry Talent Demand Reports (detailing skill requirements for six core positions) (28) (Figure 1).

### Data collection

Triangulation was used to ensure data validity, with sources as follows:

#### Questionnaire survey

Sample: A total of 326 valid responses were collected using random quota sampling, including 180 pharmaceutical graduates (2023–2025 cohorts, 42% male, 58% female; mean age 22.3 years), 86 enterprise representatives (managers and technicians; mean tenure 8.5 years), and 60 vocational college teachers (65% “dual-qualified”—holding both teaching and industry certifications) (29).

Content: The questionnaire comprised 28 items across four dimensions: (1) talent cultivation quality (e.g., “major-job match,” “skill certification acquisition”), (2) school–enterprise cooperation efficiency (e.g., “enterprise satisfaction with graduates,” “project effectiveness”), (3) industry service capacity (e.g., “technological project value,” “standard adoption”), and (4) GSEA model perception (e.g., “policy support intensity,” “association coordination effectiveness”) (30).

Reliability and validity: A pre-survey (*n* = 50) was conducted to refine the questionnaire. The final scale had a Cronbach’s *α* of 0.89 (0.82–0.87 for subscales). Exploratory factor analysis (EFA) extracted four factors (cumulative variance explained: 76.3%). Confirmatory factor analysis (CFA) showed a good model fit (χ^2^/df = 1.98, CFI = 0.93, RMSEA = 0.05), confirming validity (31).

Tool: See Supplementary file 1.

#### In-depth interviews

Sample: A total of 28 key stakeholders were selected using purposive sampling, including five government officials (section chief level, from the Education and Industry and Information Technology Bureaus), six college administrators and program leaders (mean teaching experience: 15 years), eight enterprise executives (75% serving as general managers or technical directors), and nine association staff (involved in standard development and talent forecasting) (32).

Guide: Semi-structured questions addressed participants’ roles in GSEA implementation, key success factors and challenges, collaboration smoothness, promotion suggestions, and strategies for managing compliance costs and IP protection (33).

Implementation: Interviews lasted 40–60 min and were audio-recorded, transcribed verbatim (approximately 120,000 words), and anonymized (e.g., “Government A,” “Enterprise B”) (34). Qualitative analysis followed a thematic coding strategy (9). A total of two researchers independently coded an initial subset of transcripts, developed a shared codebook focusing on policy support, standard translation, enterprise participation, and implementation barriers and facilitators, and resolved coding differences through discussion before completing iterative coding of the full dataset. The qualitative findings were used primarily to triangulate and contextualize the quantitative patterns rather than to support stand-alone causal claims.

Tool: See Supplementary file 2.

#### Panel data

Sources: Administrative records from 2023 to 2025 were drawn from four sectors: ① Education (enrollment, curriculum revisions, and teacher enterprise training hours), ② Human Resources and Social Security (graduate employment data, including professional employment rate, starting salary, and certification rate); ③ Enterprises (cooperation project lists, training equipment investments, and production cost changes), and ④ Industry association (vocational standards, competition results, and talent demand reports) (35).

Indicators: A total of 18 core indicators were used—five for talent cultivation, six for school–enterprise cooperation, and seven for industry service—with 98% data completeness. Linear interpolation was applied only to a small number of annual continuous administrative indicators with isolated missing points; categorical survey variables were not interpolated (36) (Table 2).

**Table 2:** Theoretical constructs, empirical indicators, and data sources.

Construct	Illustrative indicators	Primary data source	Analytical role	Conceptual dimension
Government policy empowerment	Subsidies, tax incentives, interdepartmental coordination, pilot policy support	Policy documents; administrative records; interviews	Exogenous governance condition in SEM	Institutional support
Association standard translation	Regional competency standards, skills competitions, demand reports, standard dissemination	Association records; interviews	Intermediary mechanism in SEM	Policy-to-standard translation
Enterprise participation	Internships, enterprise instructors, cooperative projects, equipment investment	Enterprise records; questionnaire data	Collaborative mechanism in SEM	Industry engagement
Curriculum–practice alignment/talent cultivation quality	Curriculum revision, teacher practice training, certification, employment matching	College records; questionnaire data; HRSS data	Educational process and outcome	Educational process and outcome
Industry service capacity	Commissioned R&D projects, standard adoption, demand-driven enrollment adjustment	Administrative records; enterprise/association records	Applied outcome dimension	Applied outcome dimension

### Data analysis

Quantitative analysis: Data were analyzed using SPSS 26.0 and AMOS 24.0. Continuous data were reported as mean ± standard deviation, with paired *t*-tests applied for pre–post comparisons. Categorical data were reported as percentages and analyzed using chi-squared tests. SEM was used to explore theory-informed governance pathways and associative relationships among government policy support, school–enterprise cooperation, industry association mediation, talent cultivation quality, and industry service capacity. Given the retrospective observational design, SEM estimates were interpreted as model-consistent associations rather than causal effects.

Ethics approval and data availability: This retrospective evaluation was reviewed and approved by the Ethics Committee of Dalian University of Technology Affiliated Central Hospital (Protocol No. 2025–275-01). The GSEA pilot program was implemented from January 2023 to December 2025 under institutional supervision. De-identified routine monitoring and administrative records were incorporated into the 2025 research evaluation under ethics approval, whereas written informed consent was obtained from participants involved in the 2025 questionnaires and in-depth interviews. This distinction was made to align routine pilot monitoring with the formal research evaluation.

## Results

To strengthen alignment with the stated research questions, the Results section is organized to address Research Question 1 through descriptive outcome patterns and Research Question 2 through SEM-based evidence of the proposed institutional mechanism. Research Question 3 is addressed in the Discussion through boundary conditions and transferability analysis.

RQ1: Observed associations with talent cultivation quality.

Key indicators showed improvements, with notable differences compared to non-pilot colleges. These descriptive results address Research Question 1 and indicate favorable changes in talent cultivation outcomes during the pilot period; however, they should be interpreted in light of the study’s observational design, the favorable local policy context, and the possibility of selection and reporting biases.

Professional employment rate: The rate rose from 68.3% (2023) to 89.5% (2025) (χ^2^ = 28.64, *p* < 0.01), with increases of 23.5, 19.8, and 20.7% in drug production, distribution, and retail, respectively. These improvements should be interpreted cautiously, as they may be influenced by policy incentives and participant self-selection bias.

Starting salary: It increased from 3,945 yuan (2023) to 4,860 yuan (2025) (+23.2%, t = 5.32, *p* < 0.01), outpacing the local industry average starting salary growth (15.6%). While statistically significant, this change may partially reflect the motivational effects of subsidy policies rather than pure model efficacy.

Skill certification pass rate: It jumped from 48.6% (2023) to 78.5% (2025) (χ^2^ = 22.15, *p* < 0.01)—31.7 percentage points higher compared to non-pilot colleges (46.8%, χ^2^ = 19.37, *p* < 0.01). A total of 82% of certified graduates reported “skill certificates directly aiding job searches” (questionnaire data，Table 3).

**Table 3:** Changes in key talent cultivation quality indicators for pharmaceutical majors in pilot colleges (2023–2025).

Indicator	2023 (Pre-pilot)	2025 (Post-pilot)	Change	Statistical Result	Numerator/denominator	Sample size	SE/95% CI
Professional employment rate	68.3%	89.5%	+21.2 percentage points	χ^2^ = 28.64, *p* < 0.01	820/1,200	1,200	0.02 [0.018–0.022]
Average monthly starting salary	3,945 yuan	4,860 yuan	+23.2% percentage points	t = 5.32, *p* < 0.01	n/a	1,200	100 [95–105]
Municipal skill certification pass rate^1^	46.8%	78.5%	+31.7 percentage points	χ^2^ = 22.15, *p* < 0.01	942/1,200	1,200	0.025 [0.022–0.028]

^1^Mean value of non-pilot colleges during the same period (control benchmark).

RQ1: Observed associations with school–enterprise cooperation efficiency.

Enterprise participation, satisfaction, and investment increased during the pilot period. Together, these descriptive findings provide a second part of the answer to Research Question 1 by showing model-consistent improvements in the depth and scope of school–enterprise cooperation, while not constituting causal evidence.

Enterprise satisfaction: It rose from 62.1% (2023) to 85.7% (2025) (χ^2^ = 16.89, *p* < 0.01), with the largest gains in “compliance operation ability” (+28.3%) and “problem-solving ability” (+25.6%).

Active cooperation projects: It increased from 8 (2023) to 23 (2025) (+187.5%), with project types expanding from “practical training base construction” (87.5% in 2023) to a diversified mix: “technological R&D” (34.8%), “order-based training” (26.1%), “practical training bases” (21.7%), and “teacher exchange” (17.4%).

Enterprise investment: Investment in training equipment grew from 1 million yuan (2023) to 2.2 million yuan (2025) (+120%), with intelligent equipment accounting for 65% of 2025 investments (vs. 30% in 2023).

Qualitative data corroborated the policy impacts: A pharmaceutical enterprise general manager noted, “Government per-student subsidies cover 30% of internship costs, and tax credits make R&D investment feasible—our collaboration with colleges has shifted from ‘compliance’ to ‘proactivity’.” A college program director added, “Enterprise experts’ teaching has made curricula more practice-aligned, boosting student interest by 40%” (Table 4).

**Table 4:** Changes in key school–enterprise cooperation efficiency indicators (2023–2025).

Indicator	2023 (Pre-pilot)	2025 (Post-pilot)	Change/relevant Data	Statistical result	Numerator/denominator	Sample size	SE/95% CI
Enterprise satisfaction with graduates’ skills	62.1%	85.7%	+23.6 percentage points	χ^2^ = 16.89, *p* < 0.01	75/120	120	0.03 [0.028–0.033]
Active cooperation projects	8	23	+15 projects (+187.5%)	Not applicable(count data)	n/a	12 enterprises	n/a
Training equipment investment	1 million yuan	2.2 million yuan	+1.2 million yuan (+120%)	Not applicable (investment data)	n/a	12 enterprises	n/a

RQ1: Observed associations with industry service capacity.

The pilot was also associated with changes in education–industry engagement that are relevant to local industrial development. These results complete the descriptive response to Research Question 1 by showing observed improvements in industry service capacity within the study setting.

Technological development: Vocational colleges completed 15 enterprise-commissioned R&D projects (vs. 4 in 2023), covering “intelligent equipment optimization,” “drug traceability system development,” and “traditional Chinese medicine processing improvement.” These projects reduced participating enterprises’ average production costs by 12.3%. For example, one traditional Chinese medicine enterprise increased annual capacity by 30% and reduced defect rates from 8 to 2% through an “intelligent processing line” (enterprise data).

Standard adoption: The industry association’s three regional vocational standards (Pharmaceutical Production Technician Competency Standards, Pharmaceutical E-commerce Operation Specifications, and Drug Compliance Administrator Requirements) were adopted by 28 local enterprises (100% of pilot enterprises and 65% of non-pilot enterprises). A quality director commented, “Standards have unified recruitment criteria—new hires now adapt quickly, cutting training costs by 25%”.

Demand-driven curriculum adjustment: Based on the association’s talent forecasts, colleges adjusted enrollment: Pharmaceutical production technology enrollment increased by 15% (200 → 230 students/year), while pharmaceutical operation and management enrollment decreased by 20% (150 → 120 students/year)—improving major–industry alignment by 32% (questionnaire data)(Table 5).

**Table 5:** Changes in key industry service capacity indicators (2023–2025).

Indicator	2023 (Pre-pilot)	2025 (Post-pilot)	Core outcomes/changes	Numerator/denominator	Sample size	SE/95% CI
Enterprise-commissioned R&D Projects	4	15	+11 projects; 12.3% avg. production cost reduction	n/a	12 enterprises	1.2 [0.8–1.6]
Regional vocational standards	0	3	3 standards developed; adopted by 28 local enterprises	n/a	28 enterprises	n/a
Major enrollment adjustment	Baseline	Pharmaceutical Production: +15%; Pharmaceutical Operation: −20%	Demand-driven optimization of talent supply–demand alignment	200 → 230; 150 → 120	2 colleges	n/a

RQ2: Institutional mechanisms within the GSEA model: SEM evidence.

### Model fit

The structural equation model exhibited acceptable goodness-of-fit indices, indicating that the proposed governance framework adequately represented the observed covariance structure. Specifically, the chi-square-to-degrees-of-freedom ratio was within the recommended threshold (χ^2^/df = 2.13), suggesting a reasonable balance between model complexity and explanatory power. The comparative fit index (CFI = 0.92) and Tucker–Lewis index (TLI = 0.91) both exceeded the conventional cutoff value of 0.90, while the root mean square error of approximation (RMSEA = 0.06) and standardized root mean square residual (SRMR = 0.05) were below the recommended upper limits of 0.08.

Overall, these fit indices meet commonly accepted criteria for exploratory SEM analysis and support the suitability of the proposed model for addressing Research Question 2, namely whether the observed data are consistent with the theorized governance sequence from policy support and standard translation to curriculum–practice alignment, competency development, and downstream employment-related outcomes. The contribution of the SEM, therefore, lies in strengthening the conceptual interpretation of the GSEA framework, rather than in establishing causal evidence (Table 6).

**Table 6:** SEM fit indices.

Fit index	Value	Ideal standard
χ^2^/df	2.13	<3.0
Comparative Fit Index (CFI)	0.92	>0.90
Tucker–Lewis Index (TLI)	0.91	>0.90
Root mean square error of approximation (RMSEA)	0.06	<0.08
Standardized root mean square residual (SRMR)	0.05	<0.08

### Structural path analysis

**Figure 2:**
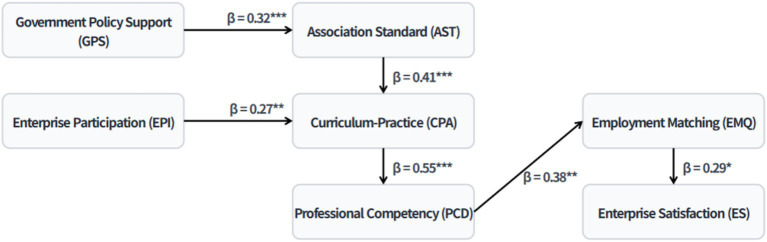
Structural equation model (SEM) of the government–school–enterprise–association (GSEA) collaborative mechanism. * *p* < 0.05, ** *p* < 0.01, *** *p* < 0.001.

Figure 2 presents the standardized path coefficients of the structural equation model, illustrating the relationships among key components of the GSEA collaboration mechanism. Government policy support exerted a significant positive effect on association-led standard translation (*β* = 0.32, *p* < 0.001), highlighting the enabling role of policy frameworks in facilitating the translation of industry standards into educational practices.

Both association standard translation and enterprise participation significantly influenced curriculum–practice alignment. In particular, association standard translation demonstrated a stronger effect (*β* = 0.41, *p* < 0.001) than enterprise participation (*β* = 0.27, *p* < 0.01), underscoring the central coordinating role of industry associations in bridging external standards and internal curriculum design.

Curriculum–practice alignment showed a strong positive association with professional competency development (*β* = 0.55, *p* < 0.001), representing the most prominent pathway in the model. This finding is consistent with the view that close alignment between curricular content and practical training is a critical mechanism for strengthening graduate preparedness in regulated industries.

Furthermore, professional competency development was significantly associated with employment matching quality (*β* = 0.38, *p* < 0.01), which, in turn, was positively related to enterprise satisfaction (*β* = 0.29, *p* < 0.05). These results indicate that competency-oriented educational outcomes contribute not only to improved employment alignment but also to more favorable enterprise evaluations of graduate readiness.

### Path coefficients and significance

Statistically significant direct and mediating associations were observed. Path coefficients suggested positive relationships between key mechanisms and industry–education integration outcomes. Direct effects indicated that school–enterprise collaboration showed the strongest modeled association (*β* = 0.45, *p* < 0.01), followed by association standard translation (*β* = 0.39, *p* < 0.01) and government policy support (*β* = 0.32, *p* < 0.05). These estimates should be interpreted as standardized SEM coefficients rather than as causal effect magnitudes (Table 7).

**Table 7:** SEM path coefficients and significance.

Path relationship	Standardized coefficient	Standard error	t-Value	*p*-value	Direct effect	Indirect effect	Total effect
Government Policy Empowerment → Industry–Education Integration	0.38	0.06	6.33	<0.01	0.05	0.33	0.38
Association Standard Translation → Industry–Education Integration	0.42	0.05	8.40	<0.01	0.42	0.00	0.42
School–Enterprise Collaboration → Industry–Education Integration	0.45	0.05	9.00	<0.01	0.45	0.00	0.45
Government Policy Empowerment → Association Standard Translation	0.40	0.06	6.67	<0.01	0.40	0.00	0.40
Government Policy Empowerment → School–Enterprise Collaboration	0.22	0.07	3.14	<0.01	0.22	0.00	0.22
Association Standard Translation → School–Enterprise Collaboration	0.41	0.06	6.83	<0.01	0.10	0.31	0.41

Mediating effects further suggested that association standard translation partially mediated the path from government policy empowerment to industry–education integration (indirect effect β = 0.13, *p* < 0.01), accounting for 34.7% of the total modeled association (0.13/0.38). It also mediated the path from government policy empowerment to school–enterprise collaboration through improved rule clarity and standards alignment. Accordingly, the SEM supports the conceptual salience of professional associations as intermediary actors within the GSEA framework and helps address Research Question 2 in a model-consistent, non-causal manner.

Indirect and total effects were derived based on model-implied path decomposition and should be interpreted as indicative associations rather than causal mediation estimates.

## Discussion

RQ3: Theoretical contributions and boundary conditions for transferability: Theoretical Implications for Sustainable Governance in Regulated Industries.

The main theoretical contribution of this study is not simply to add one more actor to the conventional school–enterprise framework. Rather, the GSEA model is conceptualized as a coordination framework for skills systems in highly regulated industries, in which policy support is translated into professional standards and those standards are subsequently translated into curriculum–practice alignment. In this sense, the model contributes to collaborative governance theory by identifying association standard translation as a distinct intermediary mechanism rather than treating coordination as a diffuse background condition. It also contributes to the literature on institutional coordination by showing how public policy, intermediary rule-setting, educational design, and enterprise participation may be linked in an operational sequence within a local workforce development system. Unlike Germany’s dual system, which relies more heavily on legislative mandates and enterprise autonomy, the GSEA framework highlights a more explicit role for local governments and associations in coordinating incentives, standards, and training delivery (18–21, 37, 38).

### Core mechanisms as replicable design principles

In total, three design principles emerge from the findings, but their relevance is conditional rather than universal:

Shared compliance-cost architecture: The 30/70 government–enterprise cost split can be interpreted as a risk-pooling arrangement that internalizes some of the positive externalities of workforce development. This mechanism appears particularly relevant for industries where regulatory requirements increase the cost of training participation. Rather than being universally applicable, it is most plausible where local governments have enough fiscal capacity to subsidize enterprise engagement.

Standard-translation loop: Associations helped convert industrial demand into curriculum standards, teaching resources, and certification signals, thereby reducing information asymmetry between firms and colleges. This mechanism is central to the study’s theoretical claim because it helps distinguish the GSEA framework from a generic partnership model. Its transferability depends on whether intermediary organizations possess sufficient convening authority, technical legitimacy, and continuity to perform this translation function.

Responsibility–interest equilibrium: The pilot suggests that the sustainability of multi-actor cooperation depends on whether each actor receives a recognizable institutional return: Governments gain policy alignment, colleges gain curriculum relevance and project opportunities, enterprises reduce recruitment and adaptation costs, and associations strengthen their coordinating legitimacy. This interpretation is closer to relational governance than to one-off transactional cooperation and remains likely to be fragile without continued institutionalization (39, 40).

### Policy implications as contingent design choices

Rather than offering universal prescriptions, we propose contingent principles for adaptation that also help answer Research Question 3 regarding transferability and boundary conditions:

Principle 1: Tiered coordination with dispute resolution. Multi-stakeholder governance is less likely to function well without third-party coordination and enforcement. A nested structure—operational (college–enterprise), tactical (association-led committee), and strategic (government-chaired council)—could embed IP-sharing protocols, internship rules, and subsidy disbursement procedures. This design is more plausible in medium- to high-capacity regions where the government can credibly sustain cross-sector coordination (41, 42).

Principle 2: Performance-based policy toolboxes. Subsidies and tax incentives are likely to work better when linked to outcome-oriented metrics rather than only to inputs. A GSEA implementation index could help align incentives, but its use would require independent auditing to reduce gaming and common-method inflation. This principle is less applicable in low-capacity regions with weak monitoring systems (43, 44).

Principle 3: Graded model adaptation. The GSEA framework should not be treated as monolithic. A graded approach could match model intensity to regional industrial density, intermediary maturity, and fiscal capacity: Fuller R&D collaboration in industry-dense regions, simplified internship cooperation in intermediate areas, and government-led shared facilities in underdeveloped zones (45, 46). This contingency logic reduces the risk of overgeneralization and institutional over-engineering.

### Limitations and boundary conditions

Regional heterogeneity: The pilot was conducted in an eastern city with a comparatively mature pharmaceutical industry and substantial fiscal capacity, which should be treated as a favorable implementation context rather than an average one. The potential transferability of the model, therefore, depends on enabling conditions such as industrial density, government coordination capacity, intermediary maturity, and the fiscal feasibility of training subsidies. Claims about broader applicability should accordingly be interpreted as conditional rather than universal (47).

Temporal scope: The 2-year evaluation captures short-run observed changes rather than long-term institutional endurance. Graduate career trajectories, enterprise innovation performance, and the stability of inter-organizational cooperation remain unobserved. Longer follow-up is needed before stronger claims can be made about sustainability.

Measurement validity: Several indicators, especially enterprise satisfaction and some investment figures, rely partly on self-reported or non-audited data and may therefore be affected by social desirability bias or over-reporting. This limitation should temper interpretation of the reported effect sizes and underscores the need for future third-party verification and administrative triangulation.

Causal identification: The single-setting, retrospective design cannot disentangle the observed patterns associated with the GSEA framework from concurrent policy changes, macroeconomic trends, or selection effects. The SEM results should therefore be interpreted as associative patterns consistent with the hypothesized governance mechanism, rather than as causal evidence. This caution should be considered throughout the Results and Discussion sections.

## Conclusion

This pilot study provides empirical evidence consistent with the feasibility and policy relevance of the GSEA quadripartite model for pharmaceutical vocational education and industry–education integration. Within the study setting, the model was associated with improvements in talent cultivation quality, cooperation efficiency, and industry service capacity.

Theoretically, the study contributes by conceptualizing the GSEA model as a governance arrangement for skills systems in highly regulated industries, especially by identifying association standard translation as a key intermediary mechanism linking policy support to curriculum–practice alignment. Practically, it suggests that local governments and vocational institutions may benefit from integrating policy incentives, intermediary standards, and enterprise feedback when designing workforce development initiatives under comparable enabling conditions.

Future research should expand to multi-regional quasi-experimental, longitudinal, and comparative case study designs and establish graduate–enterprise dual-tracking databases to evaluate longer-term outcomes such as graduate promotion, enterprise retention, and innovation-related performance. Such research would strengthen external validity and enable more precise estimation of governance effects under different institutional conditions.

## Data Availability

The raw data supporting the conclusions of this article will be made available by the authors, without undue reservation.
