# Supplementary material for: GSEA model outcomes in pharmaceutical workforce development: a retrospective pilot study (2023–2025)
Source: Front Public Health. 2026 May 26;14:1812820. doi: 10.3389/fpubh.2026.1812820 (PMC13246716; doi:10.3389/fpubh.2026.1812820)
Supplement: Supplementary file 1 [file Data_Sheet_1.PDF]

Questionnaire on the Effectiveness of the GSEA Quadripartite Collaboration Model in Pharmaceutical Vocational Education (S1)

I. Basic Information

1.Identity Type: ☐ Graduate of Pharmaceutical-related Major (2023 – 2025 Intakes) ☐ Enterprise Representative (Manager/Technician) ☐ Vocational College Teacher

2.Gender (to be filled in by graduates/teachers): □ Male □ Female

3.Age (to be filled in by graduates): □ 20–22 Years Old □ 23–25 Years Old □ Over 25 Years Old

4.Work Experience (to be filled in by enterprise representatives): □ 3 Years or Less □ 4–8 Years □ 9–15 Years □ Over 15 Years

5.Teacher Type (to be filled in by teachers): □ Dual-Qualified Teacher (holding both teaching certificates and industry-related certificates) □ Regular Teacher

6.Enterprise Type (to be filled in by enterprise representatives): □ Pharmaceutical Manufacturing Enterprise □ Pharmaceutical Distribution Enterprise □ Pharmaceutical Retail Enterprise

7.Major Studied/Taught (to be filled in by graduates/teachers): □ Pharmaceutical Production Technology □ Pharmaceutical Operation and Management □ Pharmacy □ Chinese Materia Medica □ Pharmaceutical E-commerce □ Other Pharmaceutical-related Majors

II. Talent Cultivation Quality Dimension

(Please select based on actual situations: 1 = Strongly Disagree, 2 = Disagree, 3 = Neutral, 4 = Agree, 5 = Strongly Agree)

Question No.	Question Content	1	2	3	4	5
1	The major studied/taught is highly aligned with job requirements/industry needs.					
2	The professional skills of graduates meet the practical work requirements of their positions.					
3	Graduates' professional qualities (e.g., sense of responsibility, compliance awareness) meet enterprises' expectations.					
4	Graduates have successfully obtained pharmaceutical industry-related skill certificates (e.g., GMP certification, intelligent equipment operation certificate).					
5	Skill certificates are significantly helpful for job hunting/work.					
6	Graduates adapt to their work positions quickly.					

Question No.	Question Content	1	2	3	4	5
7	The content of professional courses is closely integrated with practical industry applications.					

III. School-Enterprise Cooperation Efficiency Dimension

(Please select based on actual situations: 1 = Strongly Disagree, 2 = Disagree, 3 = Neutral, 4 = Agree, 5 = Strongly Agree)

Question No.	Question Content	1	2	3	4	5
8	Enterprises are highly satisfied with the skill level of graduates trained through school-enterprise cooperation.					
9	School-enterprise cooperation projects (e.g., training bases, joint R&D) have achieved good implementation effects.					
10	Enterprises show high enthusiasm for participating in school-enterprise cooperation.					
11	The participation of enterprise technical experts in teaching has achieved significant effects.					
12	Communication and coordination between schools and enterprises are smooth, and cooperation connection is efficient.					
13	Cooperation projects can effectively address enterprises' practical needs (e.g., talent gaps, technical difficulties).					
14	Schools can adjust their talent cultivation programs in a timely manner based on feedback from enterprises.					

IV. Industry Service Capability Dimension

(Please select based on actual situations: 1 = Strongly Disagree, 2 = Disagree, 3 = Neutral, 4 = Agree, 5 = Strongly Agree)

Question No.	Question Content	1	2	3	4	5
15	The technical R&D projects of vocational colleges have practical value for enterprise development.					
16	The vocational competency standards formulated by industry associations are instructive for talent cultivation and enterprise recruitment.					

Question No.	Question Content	1	2	3	4	5
17	The major setup of schools is consistent with the development needs of the regional pharmaceutical industry.					
18	School-enterprise cooperative R&D projects can help enterprises reduce production costs or improve production efficiency.					
19	Industry talent demand reports provide effective references for schools to adjust enrollment plans and curriculum settings.					
20	Vocational colleges provide stable skilled talent support for the development of the pharmaceutical industry.					

V. GSEA Model Perception Dimension

(Please select based on actual situations: 1 = Strongly Disagree, 2 = Disagree, 3 = Neutral, 4 = Agree, 5 = Strongly Agree)

Question No.	Question Content	1	2	3	4	5
21	The government's policy support (e.g., subsidies, tax incentives) is sufficient.					
22	Industry associations play a prominent coordinating role in the quadripartite collaboration.					
23	The skill competitions organized by industry associations have a positive effect on improving talents' skill levels.					
24	There is strong policy coordination among government departments (e.g., Education Bureau, Industry and Information Technology Bureau).					
25	The quadripartite collaboration mechanism can effectively reduce the compliance costs for enterprises to participate in school-enterprise cooperation.					
26	All participants can obtain clear benefits in the collaboration.					
27	The GSEA quadripartite collaboration model has more advantages than the traditional					

Question No.	Question Content	1	2	3	4	5
	school-enterprise dual model.					
28	You are willing to continue participating in or supporting the promotion of the GSEA quadripartite collaboration model.					

VI. Open-Ended Questions

1.What do you think is the most prominent advantage of the GSEA quadripartite collaboration model during its implementation?

2.What shortcomings do you think this model still has in terms of talent cultivation, school-enterprise cooperation, or industry services?

3.What specific suggestions do you have for further optimizing the GSEA quadripartite collaboration model?
