# Supplementary material for: GSEA model outcomes in pharmaceutical workforce development: a retrospective pilot study (2023–2025)
Source: Front Public Health. 2026 May 26;14:1812820. doi: 10.3389/fpubh.2026.1812820 (PMC13246716; doi:10.3389/fpubh.2026.1812820)
Supplement: Supplementary file 2 [file Data_Sheet_2.PDF]

In-depth Interview Outline for the GSEA Quadripartite Collaboration Model in Pharmaceutical Vocational Education (S2)

I. Basic Information of Interviewees

1. Name (Anonymity Optional): ________

2. Identity Type: □ Government Official (Education Bureau/Industry and Information Technology Bureau, etc.) □ Vocational College Administrator/Major Leader □ Enterprise Executive/Technical

3. Director □ Industry Association Staff Affiliated Institution and Position: ________

4. Duration of Participation in GSEA Collaboration and Key Responsibilities: ________

II. Core Interview Questions

(I) Role Cognition and Participation Experience

1.What are the core responsibilities of your entity in the GSEA quadripartite collaboration model? How is the actual implementation progressing?

2.Since participating in this collaboration model, what do you consider the most significant gains (e.g., policy support, talent reserve, technological breakthroughs, industry influence, etc.)?

3.During the collaboration process, do you think the role positioning of each participant (government, colleges, enterprises, associations) is clear? Are there any overlaps or gaps in responsibilities?

(II) Collaboration Mechanism and Implementation Effect

1.In your view, what is the interactive relationship among government policy empowerment, association standard transformation, and school-enterprise collaboration within the GSEA model? Which factor has the most critical impact on collaboration effectiveness?

2.From your perspective, what are the most prominent outcomes of this model in improving talent cultivation quality, enhancing school-enterprise collaboration efficiency, or strengthening industry service capabilities? Please provide examples to illustrate.

3.Does the work of industry associations in standard formulation and demand matching meet actual needs? Is the process of standard transformation (e.g., converting industry demands into curriculum standards) smooth?

(III) Compliance Costs and Intellectual Property Protection

1.What are the main compliance costs (e.g., clean workshop renovation, safety training, risk prevention and control) that enterprises face when participating in school-enterprise collaboration? Have the policy subsidies and cost-sharing mechanisms in the GSEA model effectively alleviated this pressure?

2.In school-enterprise technical R&D collaboration, are there any concerns or issues regarding the protection of intellectual property rights (e.g., pharmaceutical formulas, production processes)? Are the current protection measures effective?

3.How do you think the compliance cost-sharing mechanism and intellectual property protection mechanism should be further optimized to enhance enterprises’

enthusiasm for participation?

(IV) Collaboration Barriers and Solutions

1.What are the biggest challenges or barriers encountered during the implementation of the GSEA model (e.g., coordination efficiency, policy implementation, benefit distribution, etc.)? Please describe them in detail.

2.Regarding the above-mentioned barriers, do you think the existing solutions are effective? What room for improvement is there?

For cross-departmental and cross-entity coordination issues (e.g., unsynchronized 3.policies among government departments, disputes over the ownership of R&D achievements between schools and enterprises), what kind of coordination mechanism do you think should be established?

(V) Policy Support and Optimization Directions

1.Are there any problems with the current government policies (e.g., subsidies, tax incentives, honorary incentives) in terms of application procedures, fund disbursement, and implementation effects? How can these policies be optimized?

2.From your perspective, how should a more scientific performance evaluation system be designed to ensure the sustainable operation of the GSEA model?

3.Considering the differences in industrial foundations and financial capabilities across regions, how do you think the GSEA model should be adjusted to adapt to regional heterogeneity during its promotion?

(VI) Long-term Development and Promotion Prospects

1.Do you think the GSEA model is feasible for promotion in pharmaceutical industry clusters in other small and medium-sized cities? What are the key conditions for promotion?

2.For the long-term development of this model, which aspects do you think should be focused on (e.g., the long-term career development of graduates, the improvement of enterprises' innovation capabilities, the R&D of green pharmaceutical technologies, etc.)?

3.Beyond the existing participants, is there a need to involve other entities (e.g., research institutions, financial institutions) in the collaboration? If so, how should their roles be positioned?

(VII) Open-Ended Question

Do you have any other unmentioned opinions, doubts, or suggestions regarding the GSEA quadripartite collaboration model?
